# Supplementary material for: SBMLNetwork: A framework for standards-based visualization of biochemical models
Source: PLoS Comput Biol. 2025 Sep 22;21(9):e1013128. doi: 10.1371/journal.pcbi.1013128 (PMC12463325; doi:10.1371/journal.pcbi.1013128)
Supplement: S1 Listing — (PDF) [file pcbi.1013128.s001.pdf]

S1 Listing. TCA-cycle arrangement script. Python script that loads the TCA-cycle model, creates reaction-specific alias species, applies customized styling to compartments, species, and reactions, aligns the pyruvate dehydrogenase reaction vertically at the top, and arranges the main cycle reactions in a circular layout to generate the visualization shown in Fig.6.

```
import sbmlnetwork

model = '''
J1:  Pyr + NAD + CoA -> CO2 + NADH + AcCoA;
J2:  Oxalo + AcCoA + H2O -> CoA + Cit;
J3:  Cit -> IsoCit;
J4:  IsoCit + NAD -> CO2 + NADH + aKG;
J5:  aKG + NAD + CoA -> CO2 + NADH + SCoA;
J6:  SCoA + GDP + Pi -> CoA + GTP + Succ;
J7:  Succ + FAD -> FADH2 + Fum;
J8:  Fum + H2O -> Mal;
J9:  Mal + NAD -> NADH + Oxalo;

default_compartment is "TCA Cycle";
'''

net = sbmlnetwork.load(model)

net.create_aliases({
    'J2':  ["CoA"],
    'J4':  ["NAD", "NADH", "CO2"],
    'J5':  ["CoA", "NAD", "NADH", "CO2"],
    'J6':  ["CoA"],
    'J8':  ["H2O"],
    'J9':  ["NAD", "NADH"],
})

compartment = net.get_compartment()
compartment.set_font_color("#8BC34A")
compartment.set_font_size(200)
compartment.get_label().align_to_vertical_center()

species_list = net.get_species_list()
species_list.set_shapes("circle")
species_list.set_font_sizes(40)

core_species = net.get_species_list([
    "Pyr", "AcCoA", "Cit", "IsoCit", "aKG",
    "SCoA", "Succ", "Fum", "Mal", "Oxalo"
])
core_species.set_sizes((130, 130))
core_species.set_fill_colors("#FFEB3B")

small_molecules = net.get_species_list(["H2O", "CO2", "Pi"])
small_molecules.set_sizes((90, 90))
small_molecules.set_fill_colors("#FFEB3B")

cofactors = net.get_species_list([
    "NAD", "NADH", "FAD",
    "FADH2", "GTP", "CoA", "GDP"
])
cofactors.set_sizes((80, 80))
cofactors.set_fill_colors("#A0CED9")
cofactors.set_font_sizes(25)

reactions = net.get_reactions_list()
reactions.switch_to_curve()
reactions.set_thicknesses(22)
reactions.set_colors("#43A047")

net.get_reactions_list([
    "J2", "J3", "J4", "J5",
    "J6", "J7", "J8", "J9"
]).align_circle(
    center_at=(2200, 2800), radius=1500,
    arc_start=105, arc_end=-255
)

net.get_reaction("J1").align_vertical(
    center_at=(2200, 700), spread=800
)

net.set_size((4500, 5000))
net.draw("TCA_Cycle.pdf")
```
